# *Toxoplasma gondii* Serointensity and Seropositivity: Heritability and Household-Related Associations in the Old Order Amish

**DOI:** 10.3390/ijerph16193732

**Published:** 2019-10-03

**Authors:** Allyson R. Duffy, Jeffrey R. O’Connell, Mary Pavlovich, Kathleen A. Ryan, Christopher A. Lowry, Melanie Daue, Uttam K. Raheja, Lisa A. Brenner, André O. Markon, Cecile M. Punzalan, Aline Dagdag, Dolores E. Hill, Toni I. Pollin, Andreas Seyfang, Maureen W. Groer, Braxton D. Mitchell, Teodor T. Postolache

**Affiliations:** 1 Mood and Anxiety Program, University of Maryland School of Medicine, Baltimore, MD 20201, USA; aradford@health.usf.edu (A.R.D.); uttamraheja@gmail.com (U.K.R.); adagdag@som.umaryland.edu (A.D.); 2 College of Nursing, University of South Florida, Tampa, FL 33612, USA; mgroer@health.usf.edu; 3 Division of Endocrinology, Diabetes and Nutrition, Department of Medicine, University of Maryland School of Medicine, Baltimore, MD 21201, USA; joconnel@som.umaryland.edu (J.R.O.); pavlovichma@gmail.com (M.P.); kryan@som.umaryland.edu (K.A.R.); mdaue@som.umaryland.edu (M.D.); tpollin@medicine.umaryland.edu (T.I.P.); bmitchel@som.umaryland.edu (B.D.M.); 4 Program for Personalized and Genomic Medicine, University of Maryland School of Medicine, Baltimore, MD 21201, USA; 5 Veterans Health Administration, Rocky Mountain Mental Illness Research Education and Clinical Center (MIRECC), Rocky Mountain Regional Veterans Affairs Medical Center (RMRVAMC), Aurora, CO 80045, USA; christopher.lowry@colorado.edu (C.A.L.); lisa.brenner@va.gov (L.A.B.); 6 Department of Physical Medicine and Rehabilitation, University of Colorado Anschutz Medical Campus, Aurora, CO 80045, USA; 7 Military and Veteran Microbiome: Consortium for Research and Education (MVM-CoRE), Aurora, CO 80045, USA; 8 Department of Integrative Physiology and Center for Neuroscience, University of Colorado Boulder, Boulder, CO 80309, USA; 9 Center for Neuroscience, University of Colorado Anschutz Medical Campus, Aurora, CO 80045, USA; 10 US Food and Drug Administration, College Park, MD 20740, USA; andre.markon@fda.hhs.gov (A.O.M.); cecile.punzalan@fda.hhs.gov (C.M.P.); 11 US Department of Agriculture, Agricultural Research Service, Beltsville Agricultural Research Center, Animal Parasitic Diseases Laboratory, Beltsville, MD 20705, USA; fithian5@yahoo.com; 12 College of Medicine, University of South Florida, Tampa, FL 33612, USA; aseyfang@health.usf.edu; 13 Geriatrics Research Education and Clinical Center (GRECC), Baltimore, MD 21201, USA; 14 Mental Illness Research, Education, and Clinical Center (MIRECC), Veterans Integrated Service Network (VISN 5), VA Capitol Health Care Network, Baltimore, MD 21201, USA

**Keywords:** environment, household, genetics, heritability, infection, mental illness, parasitic infection, *Toxoplasma gondii*

## Abstract

*Toxoplasma gondii* (*T. gondii*) is an intracellular parasite infecting one third of the world’s population. Latent *T. gondii* infection has been associated with mental illness, including schizophrenia and suicidal behavior. *T. gondii* IgG antibody titers were measured via ELISA. The heritability of *T. gondii* IgG was estimated using a mixed model that included fixed effects for age and sex and random kinship effect. Of 2017 Old Order Amish participants, 1098 had positive titers (54.4%). The heritability for *T. gondii* serointensity was estimated to be 0.22 (*p* = 1.7 × 10^−8^ and for seropositivity, it was estimated to be 0.28 (*p* = 1.9 × 10^−5^). Shared household environmental effects (i.e., household effects) were also determined. Household effects, modeled as a random variable, were assessed as the phenotypic covariance between any two individuals who had the same current address (i.e., contemporaneous household), and nuclear household (i.e., the phenotypic covariance between parents and children only, not other siblings or spouses). Household effects did not account for a significant proportion of variance in either *T. gondii* serointensity or *T. gondii* seropositivity. Our results suggest a significant familial aggregation of *T. gondii* serointensity and seropositivity with significant heritability. The shared household does not contribute significantly to family aggregation with *T. gondii*, suggesting that there are possible unmeasured non-household shared and non-shared environmental factors that may play a significant role. Furthermore, the small but significant heritability effects justify the exploration of genetic vulnerability to *T. gondii* exposure, infection, virulence, and neurotropism.

## 1. Introduction

*Toxoplasma gondii* is one of the most common obligate intracellular protozoan parasites, infecting one third of the world’s population [1]. Globally, the estimated seroprevalence of *T. gondii* infection ranges from 10% to 80%, with the most common form being a latent infection resulting in small cysts formed by intracellular organisms generally found in the brain as well as in skeletal and cardiac muscles [2,3]. A powerful humoral immune response is elicited in response to these cysts [4].

Any member of the feline family can be the definitive host of *T. gondii*, with oocysts only produced in the intestine of cats. Intermediate hosts of the parasite include any warm-blooded mammal or bird. Infection occurs through direct exposure to the oocysts (including cat litter, consumption of contaminated water or unwashed vegetables, or failing to adequately wash hands after soil-contact) or by ingestion of raw or undercooked meat containing *T. gondii* tissue cysts. Once an intermediate host is infected, rapidly reproducing tachyzoites cause an acute infection, often presenting with flu-like symptoms [2]. Following acute infection, *T. gondii* persists in a slow-growing chronic form, in which bradyzoites are contained within intracellular cysts and may persist throughout the lifetime of the immunocompetent host, with occasional limited reactivation.

Chronic infection in rodents leads to behavioral changes, including nonspecific increases in exploratory behavior and specific attraction rather than aversion to a feline predator [5,6]. Seropositivity of *T. gondii* previously has been associated with schizophrenia [7], bipolar disorder [8,9,10,11], suicidal behavior [12,13,14,15,16], and possibly with depression [17,18,19] and personality disorders [20]. Risk-taking behaviors, delayed reaction time, and reduced neural processing speed have also been associated with positive *T. gondii* IgG antibody titers as well as traits of impulsivity and aggression in healthy individuals and in patients with intermittent explosive disorder [21,22]. A recent systematic review and meta-analysis has confirmed previously reported links between *T. gondii* IgG serointensity/seropositivity with traffic accidents and suicide attempts [23].

Although predictive associations increasingly confirmed links between *T. gondii* and brain and behavior, the direction of causality has not been demonstrated. For example, it is possible that impulsivity may represent a contributory cause of *T. gondii* infection (through less adequate washing of vegetables or inadequate cooking of meat) rather than a consequence of it.

If infection occurs during pregnancy, consequences to the fetus are caused by vertical transmission and can be devastating. Congenital toxoplasmosis can result in a wide array of clinical sequelae, the most severe being chorioretinitis, cerebral calcifications, hydrocephalus, pneumonia, and disseminated disease. It has been reported that primary infection during gestation was the only cause of congenital infection [24]. Spontaneous abortions and stillbirth may occur. While some babies can be asymptomatic at birth, delayed manifestations such as hearing and visual impairment, neurologic findings, and intellectual disability may develop years after birth [25].

In a host that is immunocompromised or immunosuppressed, such as in a host with HIV infection, more symptomatic reactivation of dormant infections may occur. Specifically, *T. gondii* bradyzoites convert back to tachyzoites [26] with full invasive and spreading potential, leading to acute encephalitis. Additionally, both primary infections and reactivations can lead to ocular lesions of toxoplasmosis, a cause of visual deficits [27].

The Old Order Amish of Lancaster, Pennsylvania, are a rural, primarily agricultural community, with a high seroprevalence of *T. gondii*. While exposure to *T. gondii* is required for infection, parasite factors, such as serotype, and host factors, both genetic and environmental, may play a role in susceptibility and disease course [28].

In order to better understand the nature of infectivity of pathogens, it is important to consider resilience and vulnerability to infection. Host genetic factors influence susceptibility to various infections, including mycobacterial infections [29,30] and malaria [31].

Heritability is useful for giving an approximate sense of the contributions of additive genetic effects while household analysis leads to identifying shared environmental effects. In addition, heritability is useful in suggesting whether or not there are genes to be identified and if the phenotype in question has an additive genetic component. A previous study found a high rate of chronic and recent *T. gondii* infections in fathers of congenitally infected children, suggesting that *T. gondii* infections cluster within families [32]. Yet, to our knowledge, there have been no prior attempts to investigate separate environmental household versus heritability contributions to the familial aggregation of *T. gondii* infection, and this is the first study to accomplish this aim.

## 2. Materials and Methods

### 2.1. Study Population

The study population consists of a total of 2017 Old Order Amish individuals residing in Lancaster County, Pennsylvania, who participated in a community-wide genetic population-wide ascertainment study of cardiometabolic and other health issues (the Amish Wellness Study). They represent approximately two-thirds of individuals who were targeted to participate (65.34%). They lived in 1085 individual households, also approximately two-thirds of the households invited to participate (65.1%). The number of individuals living in a household varied between one (361 participants, 361 households), two (1182 individuals, 591 households) three (246 individuals, 82 households), four (140 individuals, 35 households), five (40 individuals, 8 households) and six (48 participants in 8 households). For this *T. gondii* study, we measured plasma IgG antibody titers in these 2017 participants. This study was carried out in accordance with the recommendations of the University of Maryland Institutional Review Board (Reference number HP-00043451-24) with written informed consent from all subjects after a full explanation of the study and adequate time to ask questions. All subjects gave written informed consent in accordance with the Declaration of Helsinki. The protocol was approved by the Institutional Review Board from the University of Maryland (Reference number HP-00043451-24).

The Amish are ideal to examine heritability of *T. gondii* infection because (a) the large, well-characterized 14 generation pedigree available through the Anabaptist Genealogy Database (AGDB) [33,34] provides greater power to partition the phenotypic variance into different sources of genetic and environmental variances, and (b) the relatively high *T. gondii* seroprevalence (56%) [35] in this community provides substantial power to detect genetic effects.

### 2.2. Plasma Collection

The fasting blood draw was obtained by venipuncture either in the Amish Wellness Study mobile clinic, the participant’s home, or at the Amish Research Clinic. Whole blood, collected in heparinized tubes, was centrifuged at 5 °C at 3330–3350 rpm for 10 min. Plasma was separated and placed in Eppendorf tubes for storage at −80 °C until assayed for *T. gondii* IgG. All standard Institutional Biosecurity norms of the University of Maryland School of Medicine were respected and all personnel had training, retraining, and examination results set forth by biosafety procedures and research ethics norms listed by federal guidelines and Maryland University Institutional Review Board.

### 2.3. Laboratory Analysis

Plasma *T. gondii* IgG seropositivity and serointensity were measured by enzyme-linked immunosorbent assay (ELISA) at the University of South Florida College of Nursing Biobehavioral Lab in Tampa, Florida, USA. The samples were analyzed by ELISA that tests for levels of RH factor, which is the major tachyzoite antigen used in ELISAs (RE58371, IBL International, Männendorf, Switzerland). All the assays used standards for validation. Both a qualitative and a quantitative approach were used to define status. The qualitative approach uses a predetermined cutoff value as reported by the manufacturer of the kit. The optical density (OD) of each sample was compared to the cutoff standard value (greater than 12 IU/mL). Concentrations of <8 IU/mL were defined as negative. When the ELISA results indicated an equivocal concentration of *T. gondii* antibody (i.e., 8 to 12 IU/mL), we repeated the ELISA (*n* = 128 participants). When the second ELISA remained in the equivocal range or showed a level less than 8 IU/mL, the data were considered negative. If the second ELISA in those with an equivocal antibody concentration on the first testing showed a concentration in the positive range (greater than 12 IU/mL) the data were considered positive. For the quantitative analysis, the ODs of the standards were plotted against their concentrations using Graph Pad Prism software and a Cubit Spline method. The concentrations of the samples were then read from the standard curve. The serointensity was defined as the concentration of the IgG antibodies, statistically analyzed as log titer. 

### 2.4. Statistical Analysis

Since *T. gondii* IgG titer levels were skewed to the right, values were log transformed prior to analysis. Heritability was defined as the proportion of the total trait variance attributable to the additive effect of genes and was estimated by modeling the degree of biological association. Heritability was estimated using the maximum likelihood method with the SOLAR software package [36]. We additionally modeled household effects, also as a random variable, as the phenotypic covariance between any two individuals who had the same current address. We measured the contemporaneous (current) household effect, including parents, children, siblings, and spouses) as well as the nuclear household effect that measured the phenotypic covariance between parents and children only, not other siblings or spouses.

## 3. Results

The total number of participants who reported their occupation as farming was 364/2017 (18.0%). Table 1. shows the mean characteristics of the study sample.

### 3.1. T. gondii IgG Antibody Titers

Of the 2017 individuals with IgG antibody titer measurements, the total number of seropositive individuals was 1098 (54.4%) with more infected men (60.9%) than women (49.7%). The mean (SD) of IgG antibody titer in the sample was 48.5 (6.0) IU/mL and was slightly lower in women (45.8 IU/mL) than in men (52.2 IU/mL). There was a positive association between age and transformed serointensity, with beta (se) and *p*-value of 0.021 (0.002), *p* < 0.0001.

### 3.2. Heritability

The heritability for *T. gondii* serointensity was estimated to be 0.22 (*p* = 1.7 × 10^−8^). Heritability for *T. gondii* seropositivity was estimated to be 0.28 (*p* = 1.9 × 10^−5^). The heritability of serointensity between mothers and offspring, *h2* = 0.265 (*p* < 0.0001) was higher than the heritability between fathers and offspring *h2* = 0.158 (*p* = 0.005).

### 3.3. Household

Household effects did not account for a significant proportion of phenotypic variance in either *T. gondii* serointensity (*p* = 0.18) or for *T. gondii* seropositivity, regardless of whether heritability was included in the model (*p* > 0.05 for both). However, the nuclear household effect approached statistical significance (*p* = 0.06).

## 4. Discussion

To our knowledge, this is the first study to examine the heritability and household effects of *T. gondii* seropositivity and serointensity in any population. We found a moderate heritability of 28% for seropositivity and 22% for serointensity, and no significant effect of household (statistical trend for nuclear household, and no effect of contemporaneous household), suggesting that there were likely unmeasured non-household shared and non-shared environmental factors that may have played a significant role. Studies of the Old Order Amish provide an unparalleled opportunity to analyze family structure effects, including nuclear family and shared environmental effects. As suggested by the results of this study, the nuclear family is more important for serointensity than an individuals’ current household and is capable of capturing environmental developmental exposures to *T. gondii* oocysts and to factors that modulate non-Th1 immune responses that may be very different in quality and impact of the adult exposure of the Amish.

While the current *T. gondii* IgG seroprevalence in the United States is approximately 10%–15% [2], the seroprevalence in Old Order Amish is much higher (Boyer et al., 2011). Specifically, in our study, we found a seroprevalence of 54%. The higher rate of seropositivity in the Amish may be related to a higher exposure to food- and non-food-related risk factors [35]. This raises the possibility that heritability of personality traits, such as impulsivity, may increase the risk of exposure to *T. gondii* (e.g., through food processing and hand hygiene).

Serointensity is indicative not only of the virulence of *T. gondii*, but also of reactivation from latent infection [37]. Serointensity is influenced by the duration since infection, meaning that the higher the antibody titer, the more recent the infection and the extent to which the tissue cysts have spread [38].

For example, heritability estimates of HIV range from 32%–46% [39], and heritability of tuberculosis, from 50%–80% [40]. For malaria, another apicomplexan infection (as *T. gondii*), the heritability estimates for both maximum and overall parasite trophozoite density phenotypes for *Plasmodium falciparum* (*P. falciparum*) and *P. vivax* in a Karen population in Thailand were significantly and relatively similar to the ones we are currently presenting (*P. falciparum:* 16% and *P vivax*: 13%–15%) [41]. Similarly, the heritability of the number of the clinical episodes varied between 10% for *P. falciparum* and 19% for *P vivax*. In another study based on data from Senegal, a significant heritability has been reported for asymptomatic infections with *P. falciparum*, but not for symptomatic infections. While, in general, estimations of heritability were similar across locations, there were also large differences between locations, suggesting geographic variation of heritability. For example, in Dielmo and Ndiop (Senegal) heritability estimates for markers of asymptomatic infection with *P. falciparum* are reported as 15.6% and 16.3%, just a bit lower than for our current *T. gondii* study. Meanwhile, Gouye Kouly (also in Senegal) had a high level of heritability (57.1%), higher than in our current study. Similar to our current study the household effects were not significant [42]. A somewhat divergent study in Uganda has identified a significant 26.0% heritability of *Plasmodium* parasite density in children, but not adults, and a 5% household effect in adults, but not in children [43]. In our study, the lack of household-related environmental effects could be due to homogeneity of risk factors in the household of the Amish. It could also mean that non-household related environmental effects, for instance, occupational effects (contact with soil, animals, raw meat), could be more important than the household effect. Of relevance, eating and hand/food hygiene habits are mainly acquired in early childhood rather than the current, i.e., contemporaneous, household.

Limitations of our study include not having a full history of shared household and the exposure time. Additionally, this study was conducted in a highly specific population, and thus, the generalizability of the results might be limited. Another limitation is the inability to distinguish between the effect of transmission of infection during pregnancy and true heritability, potentially spuriously inflating the heritability effect or even producing a falsely significant heritability. Indeed, the heritability in serointensity between mothers and offspring, *h2* = 0.265 (*p* < 0.0001) was higher than heritability between fathers and offspring, *h2* = 0.158 (*p* = 0.005). Yet, the heritability between fathers and offspring was highly significant, arguing against a simple spurious association fully explained by an aftereffect of pathogen transmission of infection during pregnancy. We also did not calculate the heritability of the *T. gondii* clinical illness, rather than seropositivity. Data from the better-studied apicomplexan-caused disease—malaria—shows that heritability estimates for outpatient and inpatient, i.e., more severe disease, were of 24% and 25% respectively, with a much stronger household effect (29% and 14%, respectively) than reported for asymptomatic infection [42,43,44]. Finally, one-third of the invited population, in terms of both household number and person numbers, did not take part in the study. This could have affected the results in ways that are not fully known. We expect that it is possible that missing data could have inflated somewhat heritability estimates, as they appeared lower in males (as shown above), and males overrepresented females among those “missed” by recruitment.

As strengths of the study, the high seropositivity and serointensity, and the accuracy of genealogical data in the Amish provide unsurpassed advantages. Our heritability results provide the supportive rationale for efforts to study genetic contributions to *T. gondii* seropositivity and serointensity. Our next step is to further investigate genetic predisposition and resilience for *T. gondii* seropositivity and serointensity in the Old Order Amish, including, sequentially, candidate gene, genome-wide association studies (GWAS), and exome sequencing analyses. Additionally, specific exploration of *T. gondii* serotype and type of infection (e.g., via oocyst vs. tissue cyst), and capturing seroconversion and exposure to risk factors using longitudinal designs, may further help understand, prevent, and manage *T. gondii* infection. It is possible that genetic effects resulting in a significant heritability may also lead to increased novelty seeking (exploration) [45,46] and impulsivity [47,48], which, in turn, may increase the risk of *T. gondii* infection through increased exposure and incomplete hand hygiene or non-hygienic food preparation, known risk factors for acquiring *T. gondii*. Thus, increased heritability may be manifested not so much by vulnerability to infection when exposed to oocyst or tissue cyst, but through behavioral traits [21,49,50], such as genetic effects on impulsivity and decision making that may lead to variation in individual exposure levels to *T. gondii* infection risk factors. To our knowledge, this is the first study of combined evaluation of, and reciprocal adjustment for, *T. gondii* heritability and household effects, in contrast to several such studies for malaria parasites. Our study supports efforts to identify genetic, epigenetic, and environmental risk factors for *T. gondii* infection, in interaction, rather than in isolation, and might thus contribute to progress in identifying, and potentially reducing, vulnerabilities for *T. gondii* infection. This may lead to substantial preventative impact on negative child health outcomes (via reducing congenital toxoplasmosis), and negative adult health outcomes (perhaps by reduction in the rates of mental illness, such as schizophrenia [7], and potentially lethal consequences attributed to behavioral dysregulation linked to *T. gondii* infection (such as suicidal behavior, and motor vehicle accidents).

## 5. Conclusions

Our heritability results provide impetus for efforts to study genetic contributions to *T. gondii* seropositivity and serointensity. Our next step is to further investigate genetic vulnerability and resilience for *T. gondii* seropositivity and serointensity in the Old Order Amish, including, sequentially, candidate gene, GWAS, and exome sequencing analysis, in interaction with reported environmental risk factors for *T. gondii* in the Amish, currently being analyzed.

## Figures and Tables

**Table 1:** Demographics and heritability.

	All (N = 2017)	Men (N = 850, 42%)	Women (N = 1167, 58%)
Age, mean (SD)	44.0 (17.0)	45.5 (16.5)	42.9 (17.2)
BMI, mean (SD)	26.6 (5.0)	26.0 (4.1)	27.1 (5.5)
*T. gondii* antibody titer, IU/mL, mean (SD)	48.5 (66.0)	52.2 (63.0)	45.8 (68.0)
Log (titer), mean (SD)	2.7 (1.9)	2.9 (1.8)	2.5 (1.9)
Seropositive, N/total (%)	1098/2017(54.4)	518/850(60.9)	580/1167(49.7)
